# Microscopy of *Physcomitrella patens* sperm cells

**DOI:** 10.1186/s13007-017-0186-2

**Published:** 2017-05-10

**Authors:** Nelly A. Horst, Ralf Reski

**Affiliations:** 1 Plant Biotechnology, Faculty of Biology, University of Freiburg, Schaenzlestr. 1, 79104 Freiburg, Germany; 2 BIOSS – Centre for Biological Signalling Studies, University of Freiburg, Schaenzlestr. 18, 79104 Freiburg, Germany

**Keywords:** Development, Land plant motile sperms, Moss, *Physcomitrella patens*

## Abstract

**Background:**

Archegoniates (bryophytes, ferns and gymnosperms), such as the moss *Physcomitrella patens*, possess freely motile sperm cells (spermatozoids) which reach the egg cell via surface water. Although these motile flagellated sperm cells are a traditional botanical subject, they have not been thoroughly analysed in the flagship non-seed plant model species *P. patens*. Protocols are required to determine the behaviour of wild type sperms as a prerequisite for future research such as the characterization of mutants or factors that influence sperm number, morphology, viability and motility.

**Results:**

Here, we present protocols for the observation of fixed, as well as live sperms utilizing a standard microscope at intermediate magnifications. Fixed samples can be used for the fast assessment of sperm number and morphology. To determine functionality, the observation of live sperms is required. Protocols for determining both sperm motility and viability are provided, allowing both parameters to be distinguished.

**Conclusions:**

These step-by-step protocols are particularly useful for researchers so far not familiar with the analysis of motile gametes and are meant to aid the establishment and improvement of these analyses in order to stimulate research on spermatogenesis in the moss model species *P. patens*.

## Background

*Physcomitrella patens* is the only non-seed land plant selected as a flagship genome by the Joint Genome Institute [1]. As an early-diverging land plant it has proven invaluable for the elucidation of the origin and evolution of developmental pathways during the conquest of land by plants [2–7]. The change from an aquatic to a terrestrial habitat required adaptions such as protection against desiccation [8]. Notably, archegoniate plants, such as the bryophytes, possess freely motile sperms that require surface water for fertilization.

The use of *P. patens* as a model organism requires methods and documentation of all life stages. The development and morphology of most gametophytic and sporophytic life stages is well-described, e.g. [9–13], providing the basis for the phenotypic analysis of mutants or in response to treatments. The notable exception is the analysis of the biflagellated spermatozoids, which was rarely addressed in *P. patens* research.

Bryophyte sperms are a traditional botanical subject [14, 15] and their ultrastructure has been extensively analysed [16–18]. While the light-microscopic analysis of the sperms is documented in many moss species, e.g. [19–24], and has become a recent focus in the liverwort *Marchantia polymorpha* [25, 26], the methodology is not established for *P. patens*. We assume the lack of detailed protocols is the major obstacle preventing research on *P. patens* sperms and discuss possible reasons so far hindering the establishment of suitable protocols.

**Fig. 1:**
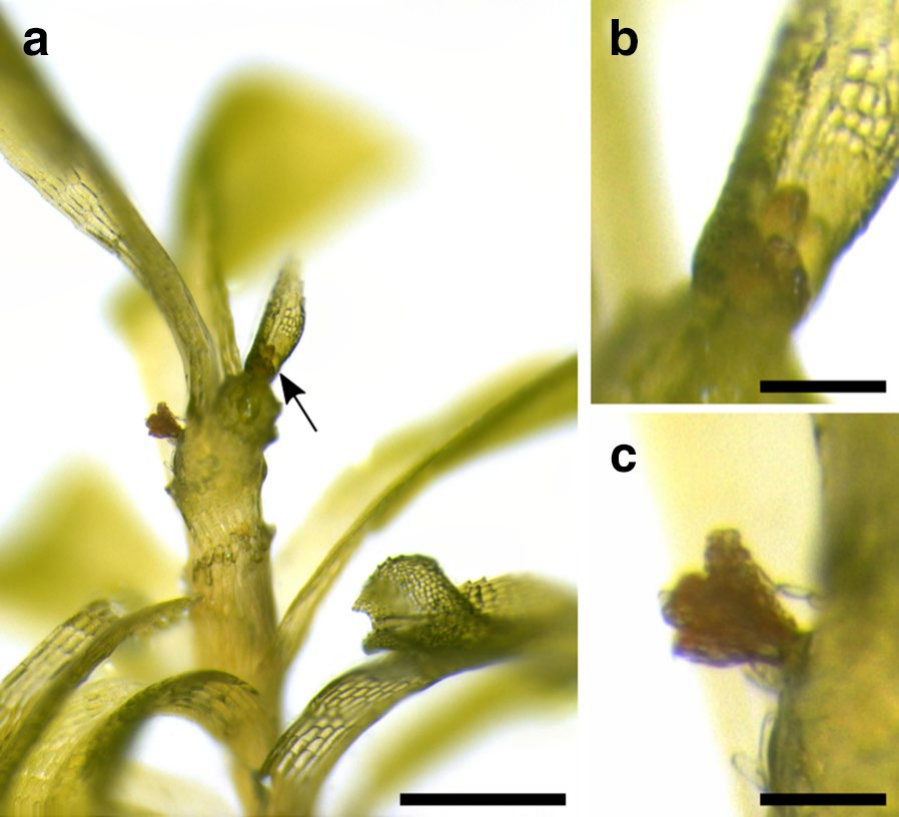
Clusters of antheridia on a *Physcomitrella* gametophore (some leaves were removed). **a** A cluster of open, empty antheridia on the stem close to the apex and a cluster of closed antheridia at the apex which is suitable for the observation of sperms (*arrow*). **b**, **c** Magnification of the clusters of closed (**b**) and open (**c**) antheridia. *Scale bars* 500 µm in **a**; 50 µm in **b** and **c**

**Fig. 2:**
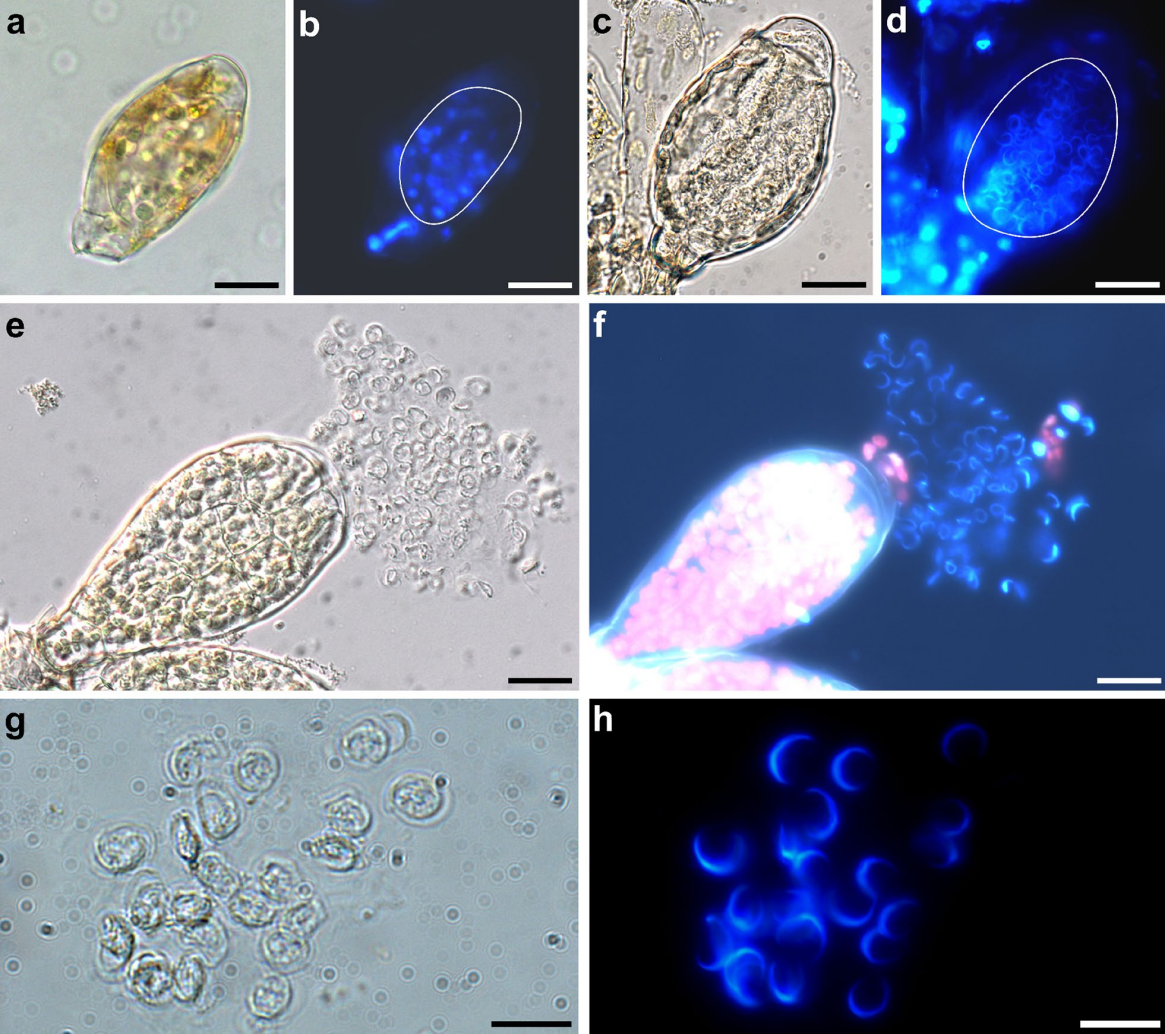
*Physcomitrella* sperms in fixed samples. **a**, **b** A developing antheridium. **c**, **d** An antheridium containing differentiated sperms, note the swollen apical cell of the antheridium. The inner space of the antheridia is indicated in *white line* in **b** and **d**. **e**, **f** A sperm mass released from an antheridium. **g**, **h** Magnified sperms. Images show the same view in bright field (*right*) and fluorescence channels. DNA was stained with DAPI. *Scale bars* 20 µm in **a**–**f**; 10 µm in **g**, **h**

At decreased temperatures and short day conditions [27] *P. patens* epidermis cells at and near the apex of the leafy gametophore reprogram into gametangia stem cells which give rise to the antheridia (Fig. 1) and later the archegonia [12]. The development of *P. patens* gametangia is described in [12]. Briefly, antheridia are multicellular structures with the inner cells developing into the spermatozoids (Fig. 2a, b). At maturity, an antheridium is an urn-shaped structure holding the sperms closed by an apical cap cell (Fig. 2c, d). The contents in the chamber of ripe antheridia are under pressure and contents of the apical cap cell become degraded, the cell becomes metastable [21]. Upon disturbance the apical cell bursts releasing the sperms (Fig. 2e–h). The sperm mass is embedded in a hydrophobic liquid/matrix allowing a passive spread of a liquid layer on the water surface [22]. Requiring surface water, they reach the archegonium and fertilize the egg cell within.

### Protocols

1. Induction of gametangia
  Prepare 9-cm vented petri dishes with about 45 ml Knop mineral medium 250 mg/L KH_2_PO _4_, 250 mg/L KCl, 250 mg/L MgSO_4_, 1000 mg/L Ca(NO_3_)_2_, 12.5 mg/L FeSO_4_ [28], supplemented with microelements (50 μM H_3_BO_3_, 50 μM MnSO_4_ × H_2_O, 15 μM ZnSO_4_ × 7 H_2_O, 2.5 μM KI, 0.5 μM Na_2_MoO_4_ × 2 H_2_O, 0.05 μM CuSO_4_ × 5 H_2_O, 0.05 μM CoCl_2_ × 6 H_2_O) [29]. Adjust pH to 5.8 with KOH. A protocol is provided in [30].
  Distribute up to twelve individual gametophores on a 9-cm petri dish. Entire gametophores are picked with flame-sterilized forceps (about 100 mm length) with fine serrated jaw (e.g. No. 10, Bochem Instrumente GmbH, Weilburg, Germany). For best growth pick gametophores from plants that have been cultivated on Knop medium as specified above for 4–6 weeks.
  Seal 7/8 of the petri dish with Parafilm M^®^ (Bemis Company, Inc., Neenah, WI, USA), and cover the remaining gap with Micropore™ Surgical Tape (3M™, Minnesota, USA). The gas exchange improves gametophore development.
  Cultivate the plants for at least five weeks in a growth cabinet or climate chamber at 22 °C, under a 16:8 h light:dark photoperiod with a light intensity of 50–70 µmol m^−2^ s^−1^ [31].
*Critical step* Prior to transferring the plates to gametangia-inductive conditions the plants need to be well-developed, i.e. gametophores should have a length of about 5 mm. The necessary pre-cultivation time is variable, depending for example on the starting material and may need to be increased for mutants with impaired gametophore development. Bigger gametophores develop gametangia faster.
  Transfer the plates to gametangia-inductive conditions, i.e. 15 °C, 8:16 h photoperiod with a light intensity of 20 µmol m^−2^ s^−1^ [27].
  Start checking for antheridia under a dissecting microscope after two weeks, clusters of antheridia of a light brown colour develop on the apex and on apical parts of the stem (Fig. 1).
*Note* When no sporophytes develop, new antheridia keep developing leading to big clusters of antheridia. Though it may appear advantageous to have many antheridia to analyse, these clusters are composed mainly of dead antheridia containing non-viable sperm. Young clusters of antheridia as pictured in Fig. 1b should be selected for analysis.
2. Fixed samples for assessment of sperm number, sperm shape, and sperms within antheridia
  Place entire gametophores into the fixative (50% v/v ethanol, 3.7% v/v formaldehyde, 5% v/v acetic acid) in a 1.5-ml reaction tube for 30 min. The volume of fixative should be at least 10× the sample volume. Remove the fixative by pipetting and replace it with water.
  Under a dissecting microscope, place a drop of water on a polylysine-coated slide (e.g. Poly-Prep Slides, Sigma-Aldrich, USA). Hold a gametophore with one pair of forceps and using another pair of forceps, brush the antheridia into the water and distribute them onto the slide.
  Let the slides dry on the benchtop overnight. The apical cells of some antheridia will rupture, releasing the sperms.
  Place a drop of phosphate-buffered saline containing 1 mg/L DAPI (4′,6-diamidin-2′-phenylindole dihydrochloride, Sigma-Aldrich) on the sample and place a coverslip on top. The rehydrated samples, including the released sperms, will stay attached to the polylysine-coated slides.
  Observe developmental stages of maturing sperm within antheridia and released sperms (Fig. 2). At the beginning of spermatogenesis (Fig. 2a, b) the nuclei are spherical (as shown after staining with DAPI in Fig. 2b), mature spermatozoids (Fig. 2c–h) have a coiled nucleus.
  Mark all visible sperms to approximate their number (Fig. 3). Adequate selection of the sample and analysing different focal planes of the same antheridium will improve the accuracy of the result.

**Fig. 3:**
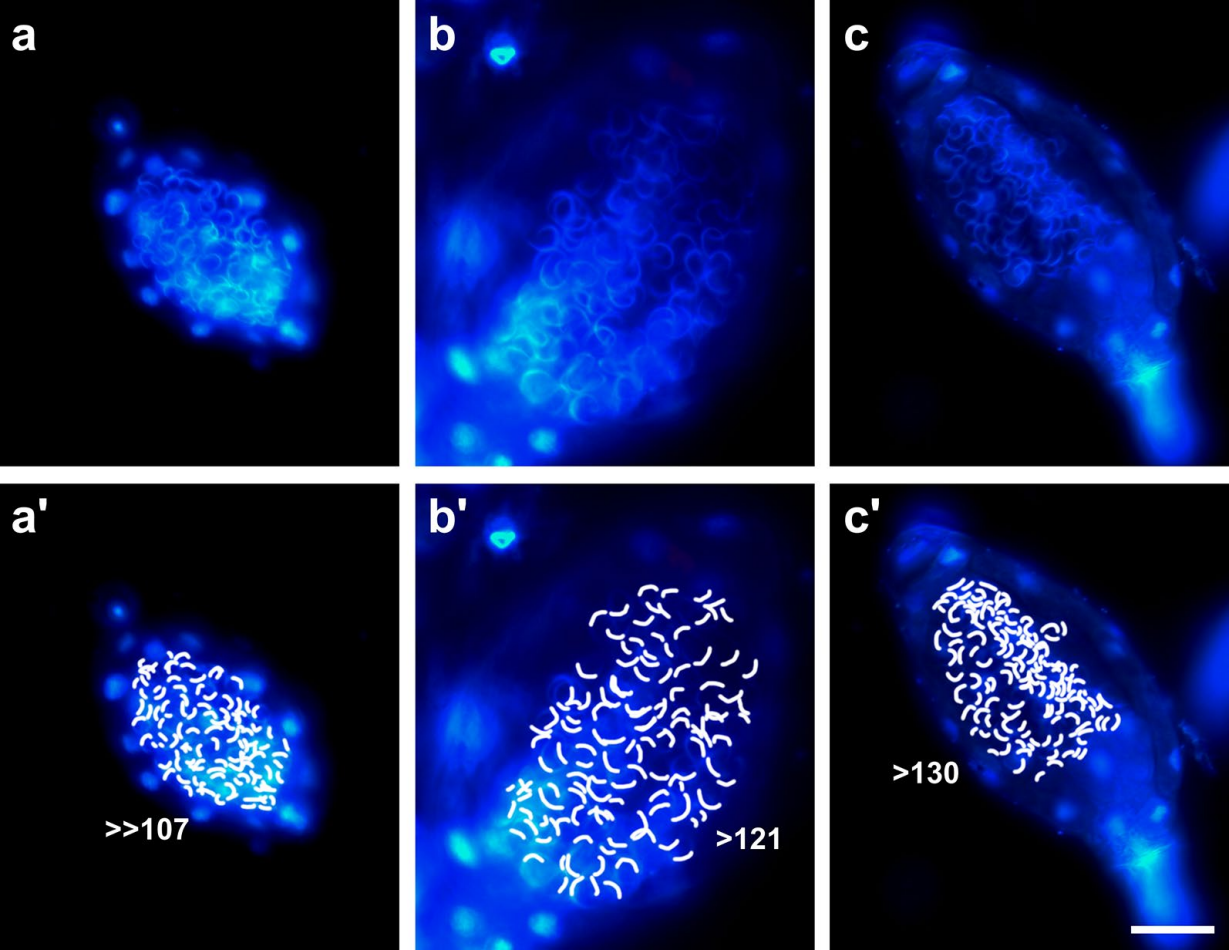
Approximation of sperm number per antheridium. Fixed samples of three antheridia (**a**, **b**, **c**) with each visible spermatozoid indicated (**a**′, **b**′, **c**′). DNA was stained with DAPI. Panel b has been reused from Fig. 2. *Scale bar* 50 µm
3. Observation of live cells for sperm motility
*Critical step* Mechanical stimulus causes the apical cell of the antheridium to burst, releasing the sperms. To ensure the antheridia are intact they need to be undisturbed prior to observation. For this, attach the bottom of the closed petri dish with double-sided tape to the benchtop next to the dissecting microscope and leave undisturbed for 24 h. The plates can be left attached to the benchtop for a couple of days until the analysis is completed.
  Place a drop of water on a microscope slide.
  Open the lid of the petri dish with the bottom staying attached to the benchtop. Close the lid between removing gametophores to avoid desiccation of the plants.
  Pick one gametophore at a time. Under a dissecting microscope hold a gametophore with one pair of forceps and using another pair of forceps, brush the antheridia into the water.
*Critical step* Immediately place a coverslip on the sample and proceed with light microscopy. The sperms are released within a few minutes once the plants are manipulated; delays will cause the sperms to disperse in the water making it more difficult to observe them and possibly missing a chance to watch an antheridium releasing the sperms.
  Using phase contrast microscopy and the 10× or 20× objective, screen the slide for sperms.
  When the apical cell of an antheridium ruptures, the bulk of the sperm mass is rapidly discharged, followed by a slow flow of the remaining sperms.
4. Staining with propidium iodide to analyse sperm viability
  Propidium iodide stains nucleic acids but does not enter intact membranes. It is therefore widely used to check for non-viable sperms [6, 23, 32, 33]. The consistency between the results obtained by phase contrast microscopy compared to the use of dyes such as propidium iodide has previously been confirmed for three moss species [23].
  Using water with 10 µg/ml propidium iodide follow the above protocol for the observation of live sperms.
  Upon detection of sperms utilizing phase contrast microscopy, switch to epifluorescence and observe propidium iodide fluorescence with a rhodamine filter set.
  Continue observing the sperms to determine their life span.
5. Microscopy
  Sperms are transparent (Fig. 4) depending on the application. An increase in contrast can be achieved by staining with DAPI or by phase contrast microscopy.

**Fig. 4:**
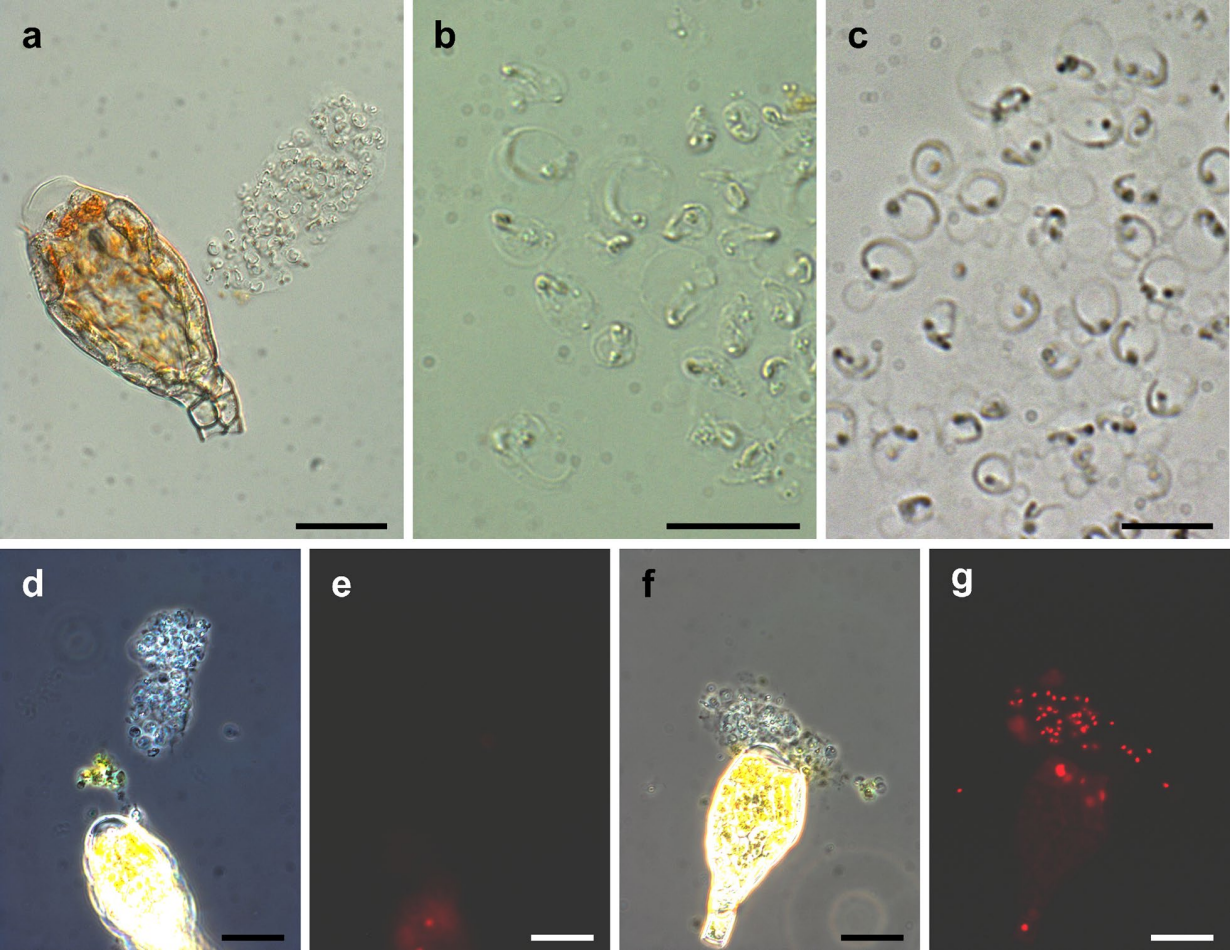
Live observation and sperm viability. **a** Bright field image of a sperm mass released from an antheridium, note that the tip cell of the antheridium is free of content due to cell rupture. **b** Bright field image of sperms. **c** Phase contrast image of sperms. Viable (**d**, **e**) and non-viable (**f**, **g**) sperms. Phase contrast (*left*) and fluorescence (*right*) images of the same view, non-viable sperms are stained with propidium iodide. *Scale bars* 50 µm in a; 10 µm in **b**, **c**; 20 µm in **d**–**g**

## Methods

Images were acquired with an Mrc5 CCD camera (Zeiss, Oberkochen, Germany) at an Olympus SZX-7 dissecting microscope (Tokyo, Japan) (Fig. 1a) and an Axioplan 2 (Zeiss). The images displayed in Figs. 2a–f, 3, 4a, d–g were acquired with a 40× objective (numerical aperture 0.75, air), the images displayed in Figs. 2g, h, 4b, c were acquired with a 100× objective (numerical aperture 1.30, oil). Immersion oil ZEISS 518 N (ne = 1.518, Zeiss) was used. Epifluorescence microscopy of DAPI or propidium iodide stained samples was performed with a BP 365 FT 395 LP 397 and a BP 546 FT 580 LP 590 filter set, respectively.

Figure 1a is composed of two images of different focal planes utilizing CombineZ (http://www.hadleyweb.pwp.blueyonder.co.uk/).

## Results and discussion

This article describes the methodology to analyse *P. patens* sperms starting with the suitable conditions to induce development of the male gametangia, the selection of appropriate samples for analysis, and easy to follow protocols for the observation of fixed and live cells with a light microscope. Presence of antheridia can be assessed in a dissecting microscope with the plants in a sealed petri dish, allowing continuing axenic cultivation until antheridia are present.

The procedure to observe bryophyte sperms described in literature is to submerge the antheridia in water, wait for the release of white sperm masses, collect these with a micropipette, and observe them in a microscope counting chamber [23, 24, 26]. Attempting to use these methods with *P. patens*, we failed to observe the release of white sperm masses under a dissecting microscope (shown e.g. in [24]) or motile spermatozoids. Paolillo [21] describes observing the release of sperms of *Funaria hygrometrica* (like *P. patens* a Funariaceae species) as “delicate and tedious” in comparison to e.g. *Atrichum undulatum, Polytrichum juniperinum*, and *Mnium affine*. Paolillo attributes this to the small size of *F. hygrometrica* antheridia, a low proportion of ripe antheridia and a particularly brief interval between disturbance by preparing the antheridia and opening of the antheridia. Furthermore, the mechanism for the expulsion of sperms varies between species: while in *A. undulatum*, *P. juniperinum*, and *M. affine* there is a fluid-filled space in the base of the antheridial chamber below the sperm mass supporting expulsion, this is absent in *F. hygrometrica* [21]. *P. patens* antheridia do not have such a fluid-filled space either, indicating a mechanism similar to *F. hygrometrica*. Therefore we hypothesized that moving the plants from the climate cabinet to the microscope already causes sufficient disturbance to release the antheridia into the water film on the gametophores. Indeed, once we started to leave the plates undisturbed for 24 h, picked one gametophore at a time, and speedily proceeded to light microscopy, we observed released motile sperms (see supplemental videos in [6]).

Bryophyte antheridia are described as containing thousands of spermatozoids [34]. The number of sperms is much lower in the *P. patens* strain used here, namely about 150–200 sperm cells per antheridium (Fig. 3). So a likely explanation for not seeing released sperm masses under the dissecting microscope is the low number of spermatozoids per antheridium and therefore the small size of the sperm masses as well as the fast release and the subsequent spread of the sperms.

Another factor is the age of the antheridia: we only observed viable sperms when looking at clusters of young antheridia. Older clusters have some closed antheridia as well; however, their spermatozoids were usually non-viable. These antheridia actually released sperms when we had not left the petri dishes undisturbed. Based on this fact we speculate that under laboratory conditions without external disturbance of the plants, a proportion of the antheridia will die without having released the sperms. In these dead antheridia the pressure in the internal space would not be maintained due to the lack of active transport of water [22] and the sperms will be squeezed out of the antheridium by the weight of the coverslip.

Overall, we suggest that the methodology developed for the analysis of other bryophyte spermatozoids is not suited for *P. patens*, requiring specific protocols as described here.

Utilizing propidium iodide to identify non-viable sperms allows a distinction between mutants affected in motility or viability, respectively. Various *Chlamydomonas reinhardtii* mutants affected in flagellar motility have been described [35], the fern *Ceratopteris richardii sleepy sperm* mutant develops slow and non-motile sperms [36]. The widely used *P. patens* Gransden 2004 strain, which was also used for genome sequencing [37], has been reported to possess reduced male fertility in comparison to the Villersexel ecotypes which may be due to a reduced number of motile sperms in Gransden 2004 plants [38]. Further research involving the various *P. patens* ecotypes is required to elucidate whether this is a variation between different ecotypes or that arose during laboratory cultivation. In previous studies the Gransden 2004 laboratory strain and the Villersexel K3 ecotype exhibited the largest amount of genetic variations between all European ecotypes tested but were able to fertilize each other [39, 40].

The spermatozoids of bryophytes are different from human sperms in morphology and development: mammalian sperms are monoflagellated with a spherical head containing the nucleus, the germ line is separated early during embryogenesis, and they are a direct product from meiosis. In contrast, the biflagellated bryophyte sperms have a coiled nucleus and develop from plant cells, requiring massive modifications such as the removal of the cell wall and the de novo establishment of microtubule organizing centres [41], and meiosis (occurring in the spore mother cell) is separated from spermatogenesis. Nevertheless, the ciliary structure was present in the last eukaryotic common ancestor [42], e.g. the 9 + 2 arrangement of the microtubule axoneme was first observed in moss spermatozoid flagella [43]. Targeted deletion of *P. patens* homologs of flagellar proteins will help to elucidate conserved functions of such proteins, with the advantage that in contrast to metazoans, such mutants can be easily vegetatively propagated and maintained as such a defect is specific to the flagellated sperms but does not affect regular plant cells.

The analysis of fixed cells provides a fast method for the general assessment of sperms, including developmental stages still within the antheridium (Fig. 2). Due to their small size, *P. patens* antheridia can be easily fixed and observed as whole-mount samples without the requirement for microscopic sections e.g. performed of *M. polymorpha* antheridia [25]. In particular the attachment of released spermatozoids to polylysine-coated slides considerably reduces the necessary preparation time for the observation of sperms.

## Conclusions

This is the first methodology article for the observation of *P. patens* sperms. Due to species-specific peculiarities of the antheridia, attempts to follow protocols used in other species were futile. The use of whole-mount samples and a standard laboratory microscope allows the fast analysis of samples without the requirement for expert knowledge of techniques that are time-consuming or not established in every laboratory. The protocol for observing fixed released spermatozoids attached to polylysine-coated slides is fast and simple and excellently suited for use in teaching. We expect these easy-to-follow protocols to aid in the establishment of the analysis of *P. patens* sperms and increase research efforts.
